# The effectiveness of nurse-led interventions to manage frailty in community-dwelling older people: a systematic review

**DOI:** 10.1186/s13643-023-02335-w

**Published:** 2023-09-30

**Authors:** Ayele Semachew Kasa, Peta Drury, Victoria Traynor, Shu-Chun Lee, Hui-Chen (Rita) Chang

**Affiliations:** 1 School of Nursing, Faculty of Science, Medicine and Health, University of Wollongong, Wollongong, Australia; 2 Department of Adult Health Nursing, School of Health Sciences, College of Medicine and Health Sciences, Bahir Dar University, Bahir Dar, Ethiopia; 3 School of Gerontology and Long-Term Care, College of Nursing, Taipei Medical University, Taipei, Taiwan; 4 School of Nursing and Midwifery, Western Sydney University, Parramatta South Campus, Parramatta, NSW Australia

**Keywords:** Nurse-led, Frailty, Education, Training, Older people

## Abstract

**Background:**

The global increase in the number of frail older people and the accompanying increase in chronic conditions underline the need to develop effective health promotion and preventive interventions for these population groups. Wide ranging of physical, psychological, and social health factors influence frailty in older people and leads to increased vulnerability to many adverse outcomes. To reverse or reduce the progression of frailty, nurses play a pivotal role in delivering health promotion and preventive interventions. The purpose of the review is to determine the effectiveness of nurse-led interventions in reducing frailty in community-dwelling older people.

**Methods:**

The following electronic databases: PubMed, MEDLINE, Web of Science, SCOPUS, CINAHL, PsychInfo, and WHO Global Index Medicus were searched until June 2022. Nurse-led, "nurse led", education, training, intervention, program, teaching, frail*, fragile*, "frailty syndrome", debility, infirmity, elder*, aged*, old*, geriatric, "community based settings", "community-based", "community setting", community were the search terms. Before data extraction, eligible articles were assessed for their methodological quality. The JBI critical appraisal checklist for reporting experimental studies was utilised to appraise the methodological quality of the studies. Data were systematically examined using a narrative review to determine the effectiveness of the intervention.

**Results:**

Of the 156 studies identified, from the search, six studies with samples ranging from 40 to 1387 older people were eligible for inclusion in the review. Two quasi-experimental studies and one Randomised Controlled Trial (RCT) showed a moderate risk of bias. The Nurse-led frailty interventions used a multi-component intervention approach across the studies. The interventions reversed frailty progression, improve physical functioning, nutritional status, and quality of life, enhance perceptions of social support, improve mental health, and reduce depression.

**Conclusions:**

Few studies have explored the effectiveness of a nurse-led intervention to decrease frailty in older people. Evaluating physical functioning, nutritional status, mental health, and quality of life in community-dwelling frail older people can contribute to developing appropriate interventions.

**Systematic review registration:**

PROSPERO ID of CRD42022348064.

**Supplementary Information:**

The online version contains supplementary material available at 10.1186/s13643-023-02335-w.

## Background

Although older people live a healthy and fulfilling life, ageing can be accompanied by declines in physical and cognitive function, which negatively impact on health status and independence [1]. Associated with these age-related declines, there are challenges to promoting the health of older people and improving their quality of life [2, 3]. One age-related challenge that compromises the health and quality of life of older people is frailty.

Although there is no consensus definition, frailty reflects a state of increased vulnerability to adverse health outcomes for individuals of the same chronological age [4, 5]. It is a geriatric clinical syndrome that results in an increased vulnerability too many adverse outcomes. Frailty in older people is influenced by a range of physical, behavioral, psychological, cognitive and social health factors [6–10]. Researchers recently designated frailty as a multidimensional concept, which encompasses losses in physical, psychological, and social functioning, and increases vulnerability to adverse health outcomes such as disability, hospitalisation, or death [1, 11]. Frail older people encounter variable health and functional life courses and frailty-associated outcomes [12, 13]. The outcomes include falls, multi-comorbidities resulting in disabilities, relocation to a nursing home, and mortality. These outcomes in frail older people are a result of the cumulative decline of multiple physiological systems and decreased resistance to stressors [14, 15]. Decreased independence and progressive disabilities challenge frail older people to maintain their activities of daily living [5]. Consequently, frailty significantly impacts on older people’s well-being and quality of life.

The global increase in the number of frail older people and the accompanying increase in chronic conditions underline the need for effective health promotion and preventive interventions [16]. The recent recognition of the multi-dimensional nature of frailty has highlighted the need for individualised multifactorial interventions targeting the physical, psychosocial, and social domains of health [17].

Prior studies had demonstrated that a health promotion intervention that detects frailty and promotes health-related behaviours was found promising to reduce frailty and also highlighted the need for high-quality studies of rigorously developed interventions [18]. Nurse-led interventions are scalable, and cost-effective, and promote positive health-related behaviours [19–21]. Nurses play a pivotal role in delivering health promotion and preventive interventions for older people [22, 23]. The nurse-led intervention improved the symptoms and lifestyle of older people and can optimise health outcomes and reduce the need for hospitalisation [24]. Studies also showed that nurse-led services that provide co-ordinated interventions were associated with fewer admission and re-admission to hospitals of individuals living with chronic conditions [25]. Generating evidence on the effectiveness of nurse-led interventions will help in reducing frailty and improve the health and well-being and quality of life of older people [26] and this in turn decreases the financial burden on frail older people.

To the researchers’ knowledge there are no prior studies investigating the impact of a nurse-led interventions on frailty status. Hence, the purpose of this systematic review is to determine the efficacy of a nurse-led intervention in reducing frailty among community-dwelling older people.

## Methods

This review is an analysis of the effectiveness of nurse-led interventional studies on community-dwelling frail older people. The review was registered at the National Institute for Health Research (NIHR) International Prospective Register of Systematic Reviews with PROSPERO ID of CRD42022348064 and reported using the Preferred Reporting Items of Systematic Reviews and Meta-Analysis (PRISMA) checklist (Additional file 1). In addition, PRISMA Flow chart was utilised to demonstrate the study selection process [27].

### Searches

A comprehensive search of electronic databases from February 20 to June 30, 2022, was undertaken. The search was conducted using PubMed, MEDLINE, Web of Science, SCOPUS, CINAHL, PsychInfo, WHO Global Index Medicus databases and registries. The following search terms were used: nurse-led, "nurse led", education, training, intervention, program, teaching, frail*, fragile*, "frailty syndrome", debility, infirmity, elder*, aged*, old*, geriatric, "community based settings", "community-based", "community setting", community. Google Scholar was used to identify additional grey literature sources. In addition to searching the electronic databases, a secondary search using the list of references of the identified articles was undertaken to identify any additional articles. Search strings were developed using "AND" and "OR" Boolean operators. The full search strategies of this review are indicated as an appendix (Additional file 2).

### Study inclusion and exclusion criteria

Original nurse-led intervention studies, quantitative interventional studies with or without a control group, focused on measuring the impact on frailty for community-dwelling older people were included. Restrictions on the date of publication for studies were not set, and the search was done until June 30, 2022. The review was restricted to studies conducted on community-dwelling older people 60 years or over and published in English language. This review included studies that reported the effect of a nurse-led intervention on community-dwelling frail older people.

### Types of participants and context

Community-dwelling older people 60 years or over [28, 29].

### Type of interventions and outcome of the study

In this study, frailty was considered as an impairment in physical, psychosocial and cognitive functions. A nurse-led intervention was defined as an intervention designed for the community-dwelling frail older people which was led and implemented by nurses. The review considered nurse-led interventions targeting community-dwelling frail older people 60 years or over. The outcome of the study is the effects of nurse-led interventions on the frailty and associated health outcomes as measured by validated measurement instruments.

### Study quality assessment

The selection process was conducted by the primary author (ASK) based on titles, abstracts, and a full test from databases, saved potentially eligible studies in Mendeley. Then, the identified articles were further screened to determine their relevance to the research question. This screening was conducted independently by two reviewers (ASK and HCC). The Joanna Briggs Institute (JBI) [30–32] critical appraisal tool was adopted to assess the methodological quality and risk of bias. A score was assigned for each item from zero for "No" or "Unclear" responses and a score of one for a "Yes" response. The scores of the items for each study were summed to obtain a total quality score. Quality of the studies were then classified into three categories as low-quality (high risk of bias) when the quality appraisal score ranged from 0 to 4, moderate quality (moderate risk of bias) from 5 to 7, and as high quality (low risk of bias) from eight and above. Studies having high and medium quality were included in the final review [33, 34]. Disagreements between reviewers were resolved through discussions or further discussion with a third reviewer (SCL).

### Data extraction strategy

A standardised data extraction form was developed. Using this form, two reviewers independently extracted relevant data from eligible articles (ASK and HCC). The specific details were extracted including authors of the study, year of publication, county, sample, setting, age, measurement tool, type of intervention, duration of intervention, and outcomes identified. Discrepancies in the data extraction were resolved through discussion with the other authors.

### Data synthesis and presentation

The synthesis without meta-analysis (SWiM) guidelines for systematic reviews [35] was used to guide data synthesis. The SWiM reporting guideline is intended to guide reporting when no meta-analysis has been performed [35]. Meta-analysis was not used in this review because of the heterogeneity in the design, methods, measurements, and interventions utilised in the primary studies. Data from eligible studies were extracted, assembled, and presented in tables. The extracted data were systematically examined and reviewed to determine the effectiveness of a nurse-led intervention in reversing or reducing the frailty of older people. Moreover, taking the generalisability of the research into consideration, the limitations of each study, and recommendations made by the studies synthesised and described.

## Results

### Review statistics and characteristics of the studies

**Fig. 1:**
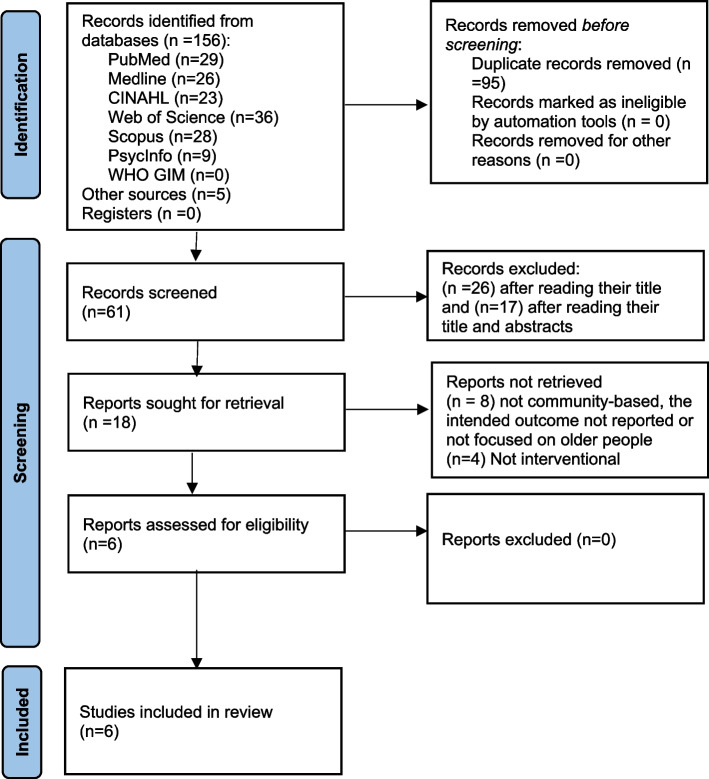
PRISMA Flow diagram showing a selection of studies, 2022

The researchers reviewed the retrieved studies and excluded 95 duplicates. The full texts of the remaining 61 studies were reviewed. Forty-three studies were excluded after reading their titles and abstracts. Twelve studies were excluded as they were not community-based, the intended outcome was not reported, not being focused on older people, or not being interventional (Additional file 3). Finally, of the 156 studies identified in the search strategy. Six studies were eligible for inclusion in the final review (Fig. 1).

Three quasi-experimental [1, 36, 37] and three randomised control trial [38–40] studies were included. The studies were conducted in Canada [38, 39], South Korea [1, 37], the Netherlands [36] and Singapore [40]. The sample size of the study participants ranged from 40 [37] to 1387 [36] with a total sample size of 2297. The studies were conducted between 2006 and 2022. The minimum age of the study participants was from 60 years old [36] to 65 years old [1, 37, 38, 40] to 75 years old (39). Two studies [38, 39] employed an adapted version of the model of vulnerability, and one employed the frailty model [37] and one adopted the multi-dimensional concept of frailty as their theoretical framework [1]. Two studies [36, 40] did not report what theoretical model was utilised to guide their studies.

The definition and measurement of frailty varied across studies, which can make it difficult to compare and generalise findings. The studies also utilised different measurement instruments. Frailty is typically assessed using a combination of physical, functional, and cognitive measures, and there is no gold standard for its diagnosis. However, many studies use established frailty scales, such as the Fried phenotype [41, 42] or the Clinical Frailty Scale [43, 44], to assess frailty. Further clarification is needed on how improvements in frailty were assessed. The Cardiovascular Health Study Frailty Index (CHS-FI) [37], Groningen Frailty Indicator (GFI) [36], and Short Physical Performance Battery (SPPB) [37] were some of the instruments utilised in the studies (Table 1).

**Table 1:** The study characteristics of the included studies, 2022

Author/year/country	Age (years), sample size	Method/design	Eligibility criteria	Theory/framework	Assessment tools	Type of intervention and setting	Frequency and duration (total sessions)	Main outcomes	Limitations	Recommendations
(Ha and Park, 2020 [37]), South Korea	≥ 65, *n* = 40	Quasi-experimental pretest–posttest	Pre-frail and registered with a seniors’ centre	Frailty model	CHS-FI, ESSI, GAS, GDSSF-K, K-CHAMPS, MNA, SPPB	A group intervention and individual goal setting compared to the control group at the community senior centre	2 sessions per week × 12 weeks (Total 24 sessions)	Statistically significant improvements in CHS-FI, GS, physical function and activity, nutritional status, and depression No significant findings: ESSI	Single site, short-term follow-ups, not mixed method	Long-term follow-up studies and additional statistical analyses needed
(Markle-Reid, Browne and Gafni, 2011 [38]), Canada	≥ 65, *n* = 210	Single-blinded RCT Three trials: Trail II: 109 Trial III: 101	Competent to give informed consent and competent in English (or with an interpreter)	An adapted version of the model of vulnerability	CES-DS, CQ, FSR, Kessler-10, HSSUI SF-36, MFES, REEN-II, RNLI, POMA, PRQ-85, SIS-16, SMMSE, SPMSQ,	Nurse-led HPDP interventions in in-home settings compared to usual home care	1 session per month × 6 to 12 months^a^ (Total 6 to 12 sessions)	Statistically significant improvements in HRQOL	Low response rate	More studies needed to evaluate the effectiveness of additional nurse-led HPDP interventions in other contexts and settings
(Markle-Reid et al*.* 2006 [39]), Canada	≥ 75, *n* = 288	Two-armed, single-blind RCT (Trial I: 288)	Existing and newly referred for personal support services through CCAC	An adapted version of the model of vulnerability	CES-DS, CQ, HSSUI, MOS (SF-36), PRQ-85, SPMSQ,	Proactive Nursing HPDP Interventions in in-home settings compared to usual home care	1 session per month × 6 months (Total 6 sessions	Statistically significant improvements: mental health functioning, depression, and perceptions in social support	The study undertaken in a well-developed urban region. The results may not be transferable to recipients of home care services living in rural areas or other environments	Home-based nursing health promotion proactively provided should be expanded to enhance the quality of life of frail older people
(Marcus-Varwijk et al*.* 2020 [36]), Netherlands	≥ 60, *n* = 1387	Quasi-experimental	Frailty, overweight, or smoking	The active ageing model, The life course perspective and Transtheoretical model	BMI, BP, GFI, FF, IM-E-SA, SrHS, WC,	Health promotion to the intervention group compared to the care-as-usual group in-home care settings	20 to 30 min per month × 12 months (Total 12 sessions)	There is no significant improvement in the GFI measure and other health related behaviour Self-reported health status in the intervention group was found good (not significant)	Control group was compiled after start of the study Reasons for loss to follow-up not mentioned Study participants were not blinded	Further research recommended to understand and evaluate nurse-led health promotion and preventive interventions targeting frail older people using diverse research designs
(Song and Boo, 2022 [1]), South Korea	≥ 65, *n* = 126	Quasi-experimental	Ageing, living alone, and prefrail or frail	The Multidimensional concept of frailty	FI, MOSSSS, SAS, TUG	Physical exercise, cognitive training, and nutrition and disease management Education compared to the control group at community senior centre	1 time per week × 12 weeks (Total 12 sessions)	Statistically significant improvements: frailty index, depression, and increased social support and social activity	Non-equivalent control group. Intervention duration and intensity may not have been sufficient to modify physical function	Strategies for disseminating sustainable nurse-led multicomponent interventions should be developed for community-dwelling older people living alone
(Ng et al*.* 2015 [40]), Singapore	≥ 65, *n* = 246	Parallel group RCT	Ageing, able to ambulate without personal assistance and living at home	NES	ADL, BMI, CHS, GDS, IADL, MMSE	Nutritional supplementation, cognitive training Physical training, combination treatment and usual care to the control group at community senior centre	2 times per week × 12 weeks^**b**^ (Total 24 sessions)	Statistically improvements in frailty score There were no major differences in self-reported hospitalisations, falls, and dependency in activities of daily living	Sample characteristics of Chinese older adults relatively younger age, good physical and cognitive functioning was noted	Identify prefrail and frail older people in the community and intervene effectively to reduce levels of frailty

*ADL* activities of daily living, *BMI* body mass index, *BP* blood pressure, *CCAC* Community Care Access Centre, *CES-DS* Center for Epidemiological Studies in Depression Scale, *CHCO* Community Health Consultation Offices for Seniors, *CHS-FI* Cardiovascular Health Study Frailty Index, *ESSI* ENRICHD Social Support Instrument, *FF* falls and fractures, *FI* Frailty Index, *FSR* Falls Surveillance Report, *GAS* Goal Attainment Scale, *GDS* Geriatric Depression Scale, *GDSSF-K* Geriatric Depression Scale Short Form-Korea Version, *GS* Grip strength, *GFI* Groningen Frailty Indicator, *HPDP* health promotion and disease prevention, *HRQOLSF-36* Health related quality of life, *HSSUI* Health and Social Services Utilization Inventory, *IADL* Instrumental activities of daily living, *IM-E-SA* INTERMED for the Elderly Self-Assessment scores, *K-CHAMPS* Korean version of the Community Healthy Activities Model Program for Seniors Questionnaire, *MMSE* Mini Mental State Examination, *MNA* Mini Nutritional Assessment, *MOS-SF 36* Medical Outcome Study Short Form (SF-36), *MOSSSS* Medical Outcomes Study Social Support

Scale, *NES* NOT EXPLICITLY STATE, *PRQ-85* Personal Resource Questionnaire-85, *RCT* randomized controlled trial, *REEN-II* Risk Evaluation for Eating and Nutrition, Version II, *RNLI* Reintegration to Normal Living Index, *SAS* Social Activity Scale, *SIS-16* Stroke Impact Scale-16, *SPMSQ* Short Portable Mental Status Questionnaire, *SPPB* Short Physical Performance Battery, *SrHS* Self-reported health status, *TUG* Timed Up and Go test, *WC* waist circumference

^a^an intervention duration varying between 6 and 12 months

^b^Nutritional supplements were taken daily for 24 weeks, after 12 weeks participants attended fortnightly 2-h “booster” sessions on cognitive training

Most of the studies included in this review were fully funded. Ajou University College of Nursing [1], the Dutch Organization for Health Research and Development [36], the National Medical Research Council [40], and the Ontario Ministry of Health and Long-Term Care, Health Research Personnel Development Fund [38] were the sources of funding. One study did not receive funding [37] and another study did not report their funding source [39].

### Study quality assessment

According to the critical appraisal, each paper was graded low to high risk of bias. The quality appraisal result showed that two of the quasi-experimental studies (Table 2) and one of the Randomised Control Trial (RCT) studies (Table 3) showed a moderate risk of bias. The rest studies demonstrated low risk of biases.

**Table 2:** Critical appraisal result of the studies using quasi-experimental study design, 2022

Included articles	Criterion No (items included to appraise quasi-experimental studies)										
	1	2	3	4	5	6	7	8	9	Raw %	Risk
Jiyeon Ha and Yeon-Hwan Park, 2020 [37]	✓	✓	*	✓	✓	✓	✓	✓	✓	88.9	Low
Song MS. and Boo S, 2022 [1]	✓	✓	*	✓	✓	✓	✓	*	✓	77.8	Moderate
Varwijk M. et al. 2020 [36]	✓	X	*	✓	✓	✓	✓	✓	✓	77.8	Moderate

√ = yes, *X* = no, * = unclear, *¥* = not applicable

Criterion No. 1: Is it clear in the study what is the ‘cause’ and what is the ‘effect’? No. 2: Were the participants included in any comparisons similar? No. 3: Were the participants included in any comparisons receiving similar treatment/care, other than the exposure or intervention of interest? No. 4: Was there a control group? No. 5: Were there multiple measurements of the outcome both pre and post the intervention/exposure? No. 6: Was follow-up complete, and if not, was follow-up adequately reported? No. 7: Were the outcomes of participants included in any comparisons measured in the same way? No. 8: Were outcomes measured in a reliable way? No 9: Was appropriate statistical analysis used?

**Table 3:** Critical appraisal result of the studies using RCT study design, 2022

Included articles	Cri**terion no.** (items included to appraise RCT studies)														
	1	2	3	4	5	6	7	8	9	10	11	12	13	Raw %	Risk
**Markle R. et al. 2006** [39]	✓	✓	✓	*	*	✓	✓	✓	✓	✓	✓	✓	✓	84.6	Low
**Markle R. et al. 2011** [38]	*	*	✓	✓	*	*	✓	✓	✓	✓	✓	✓	✓	69.2	Moderate
**Tze Pin Ng. et al. 2015** [40]	✓	✓	✓	*	*	✓	✓	✓	✓	✓	*	✓	✓	76.9	Low

√ = yes, *X* = no, * = unclear, *¥* = not applicable

Criterion No. 1: Was true randomization used for assignment of participants to treatment groups? No. 2: Was allocation to treatment groups concealed? No. 3: Were treatment groups similar at the baseline? No. 4: Were participants blind to treatment assignment? No. 5: Were those delivering treatment blind to treatment assignment? No. 6: Were outcomes assessors blind to treatment assignment? No. 7: Were treatments groups treated identically other than the intervention of interest? No. 8: Was follow-up complete, and if not, were strategies to address incomplete follow-up utilized? No 9: Were participants analysed in the groups to which they were randomized? No. 10: Were outcomes measured in the same way for treatment groups? No. 11: Were outcomes measured in a reliable way? No. 12: Was appropriate statistical analysis used? No. 13: Was the trial design appropriate?

### Intervention characteristics of the included studies: quantitative synthesis

Studies in this review employed a range of strategies to implement a nurse-led intervention. All the studies were multi-component interventions. Most studies implemented an intervention using face-to-face activities and, two studies [37, 39] utilised telephone support as the intervention strategy. The telephone support was provided in six rounds for 10–20 min per session, once every 2 weeks [37] and lasted at least 10 min that lasted for six months of follow-up [39]. Three of the studies included physical exercise as a component of the intervention [1, 37, 40] and three of the studies included nutritional education [1, 37, 40]. Cognitive education and training were utilised as an intervention in two studies [1, 40]. Nutritional supplementation was included in one of the studies [40]. Two of the studies described the training that the nurses delivering the interventions completed prior to implementing the intervention [36, 39]. Nurse training comprised of instructions on the measurement instruments; theoretical frameworks; and motivational interviewing skills [36]. The baseline and follow-up outcome assessments from the participants were obtained by trained nurses [39]. No other studies mentioned providing training for the nurses delivering the intervention [1, 37, 38, 40]. The intervention utilised planned home visits, provision of frailty education, counseling to group-based physical exercise [37]. Duration of the interventions ranged from 12 weeks [1, 37] to 12 months [36] (Table 4).

**Table 4:** Summary table on the type of intervention and main outcomes of the studies, 2022

Author/year/country	Participant characteristics	Type of intervention	Outcomes
(Ha and Park, 2020 [37]), South Korea	• Older people ≥ 65 years • Registered with a seniors’ centre • *n* = 40: 20 participants in the intervention group and 20 participants in the control group	• PNIF 2 times per week × 12 weeks (Total 24 sessions) • 6 rounds of telephone support × 10–20 min per session, once every 2 weeks • Did not state whether assessors were blinded to the participant’s group allocation • Two sessions: Session 1 and Session 2 were given for 12 weeks in consideration of individuals’ level of health, preference, and needs Session 1: Exercise and physical activity 2 times per week × 12 weeks • Upper and lower limb resistance exercises • The routine was repeated for 30 min • Range of motion exercises as a cool-down exercise Session 2: Nutritional and psychosocial interventions • Nutrition education and counselling tailored to individuals’ health status, chronic conditions, diet, household structure, and living environment • Education on mental health, such as depression relief and stress management • The intervention was provided once per week for approximately 20 min per session for 12 weeks • Data were collected using self-administered or face-to-face interviews three times: screening test, pre-test, and post-test	• CHS frailty index decreased from 1.45 to 0.70 in the intervention group but increased from 1.25 to 1.80 in the control group *(P* < *0.001)* • CHS frailty index scores range from 0 to 5 • The intervention group’s average SPPB score increased from 10.30 to 10.90 (SD = 1.52), while the control group’s score decreased from 9.70 to 9.10 (SD = 1.94) *(P* < *0.007)* • SPPB scores range from zero to 12 possible points • MNA score increased after the completion of the program in both the intervention group and the control group by 2.58 (SD = 2.42) and 0.57 (SD = 2.07), respectively *(P* = *0.009)* • MNA score had a maximum total of 30 points • The intervention group’s GDS-15 score was 0.9 lower than that of the control group *(P* = *0.018)* • No statistically significant between-group differences in ESSI *(P* = *0.779)* • The GDS-15 score ranges from 0 to 15 • No cost-effectiveness analysis was reported
(Markle-Reid, Browne and Gafni, 2011 [38]), Canada	• Older people ≥ 65 years with a range of chronic health conditions (*n* = 210) • Frail older people were eligible for home care services regardless of any level of disease severity, and other co-morbidities • Mentally competent (or with a substitute decision maker available) and competent in English (or with an interpreter available)	• Multi-component, HPDP programmes for 6 or 12 months^a^ • 1 × per month home visits; comprehensive in-home assessment of known risk factors for frailty • Linkage and referral to health and social services • Visits by a nurse or with other^b^ health professionals • Did not state whether assessors blinded to the participants’ group allocation	• HPDP intervention group had greater improvement in HRQOL and reductions in frailty compared to the usual home care • SF-36 scores range from 0 (worst) to 100 (best) • Clinically improvement in physical functioning mean score 5.87 to 15.73 *(P* = *0.009)* • Statistically significant reduction in depressive symptom scores 2.72 *(P* = *0.022)* • Significant improvement in social support score 5.26 *(P* = *0.009)* • Cost-effectiveness analysis was conducted, and the cost analysis showed that even when the costs of the HPDP interventions were included in the total cost, there was no difference in the total per-person cost of health services
(Markle-Reid et al. 2006 [39]), Canada	• Older people ≥ 75 years (*n* = 288), newly referred to and eligible for personal support services through the Community Care Access Centre (CCAC)	• Nurses visited the home over a 6-month period • One telephone contact over the 6-month follow-up • Conducted initial and ongoing health assessments, identified, and managing risk factors for functional decline (e.g., depression, dementia, polypharmacy, and co-occurring illnesses) and provided health education regarding healthy lifestyles, and the management of chronic illnesses using a participatory approach • Data were collected at baseline (T1); and at 6 months following randomization (T2) • Did not state whether assessors blinded to the participants’ group allocation	• Intervention resulted in better mental health functioning 2.61 *(P* = *0.011)*, reduced rates of depression 1.17 *(P* = *0.022)*, and enhanced perceptions of social support 2.30 *(P* = *0.009)* • An increase in role functioning related to emotional health and an increase in mental health functioning for the intervention group compared to a decrease in the usual care group • Cost-effectiveness analysis was conducted, and there was no statistically significant difference between the two groups in the mean cost of all types of health and social services
(Marcus-Varwijk et al. 2020 [36]), Netherlands	• Community-dwelling older people ≥ 60 years (*n* = 1387)	• Nurse-led consultation by a community health nurse • Consultations was provided in the areas where older people living • Nurse conducted a comprehensive health and well-being assessment, offered tailored advice and referred to other healthcare professionals • Data from the intervention and care-as-usual groups were obtained at baseline (T1) and at a 1-year follow-up (T2) • Did not state whether assessors blinded to the participants’ group allocation	• Intervention group had significantly higher median scores on the GFI of 3 *(P* < *0.01)* • The GFI score ranges from 0 to 15 • Within the intervention group an increase of 6% of older people, who rated their health status as ‘good’ was found between baseline and 1-year follow-up and this change was not statistically significant • CHCO-intervention does not improve health related behaviour measured after 1 year follow-up • The intervention group had showed a higher physical morbidity 1.73 *(P* < *0.001).* However, there was no statistical differences in psychological morbidity 1.06 *(P* = *0.22)* • No cost-effectiveness analysis was reported
(Song and Boo, 2022 [1]), South Korea	• Older people ≥ 65 years or older (*n* = 126), with low SES, prefrail or frail living alone in the community	• Multicomponent interventions • Exercise, cognitive training, and education on nutrition • 1 time per week × 12 weeks (Total 12 sessions) • The researchers who collected and analysed the data were blinded to the participants’ group allocation • Data were collected at three time points: pre-intervention (T0), postintervention (T1), and at the 12 weeks follow-up (T2)	• Non-significant reduction in frailty score from pre-intervention (T0) to 12 weeks follow-up (T2) -2.08 *(P* = *0.45)* • FI-28 score ranges from one to 28 • Significant improvements in levels of depression − 1.56 *(P* = *0.012),* social activity 1.09 *(P* = *0.002),* and social support 6.89 *(P* = *0.005)* from pre-intervention (T0) to post-test up to 12 weeks follow-up (T2) • No cost-effectiveness analysis was reported
(Ng et al*.* 2015 [40]), Singapor	• Older people ≥ 65 years identified through door-to-door open invitation • Able to ambulate without personal assistance and living at home • *n* = 246 participants: 49 in the nutrition supplementation group, 50 in cognitive training, 48 in physical training, 49 in combination, and 50 in the control group	Physical exercise (PE) • Conducted by a qualified trainer and tailored to individual abilities • 90 min duration, on 2 days per week for 12 weeks followed by 12 weeks of home-based exercises • Participants performed the exercises in groups of 8 to 10 Nutrition Intervention (NI): • Each participant provided with several supplements: iron and folate, vitamin B6, vitamin B12, calcium, and vitamin D taken daily for 24 weeks Cognitive Training (CT): • In first 12 weeks, participants attended 1 per week × 2 h session of CT • For the subsequent 12 weeks, participants attended 1 bi-monthly × 2 h ‘booster’ sessions Combination intervention (CI): • PE, NI, and CT • Outcome assessments were performed at baseline (T0), 3 months (T1), 6 months (T2), and 12 months (T3) by assessors who were blinded to the participants’ group allocation	• Frailty scores were reduced in all groups over 12 months • CHS frailty index scores range from 0 to 5 • Compared with the control group, nutritional and cognition intervention were almost 3 times more likely of frailty reduction in the intervention group, *2.98 (1.10–8.07) (P* < *0.01)* • Physical intervention was associated with 4 times higher odds of frailty reduction, *4.05 (1.50–10.8) (P* < *0.01)* • Combination intervention was associated with the highest odds of frailty reduction, *5.00 (1.88–13.3) (P* < *0.01)* • No cost-effectiveness analysis was reported

*CHCO* Community Health Consultation Offices for Seniors, *CHS* Cardiovascular Health Study, *CI* combination intervention, *CT* cognitive training, *FI* Frailty Index, *GDS* Geriatric Depression Scale, *MNA* Mini Nutritional Assessment, *ESSI* ENRICHD Social Support Instrument, *GFI* Groningen Frailty Indicator, *NI* nutrition intervention, *PE* physical exercise, *PNIF* person-centred nursing intervention program for frailty, *SF* Short Form Survey, *SPPB* Short Physical Performance Battery

^a^An intervention duration varying between 6 and 12 months

^b^Physiotherapists, occupational therapists, social workers, dietitians, and speech language pathologists

To measure the effect of nurse-led intervention, the studies followed their study participants at different time points. The studies that used telephone support ranged from 12 weeks [37] to 6 months [39].

### Nurse-led intervention settings

A nurse-led intervention at a community senior centres was undertaken in three studies [1, 37, 40]. Whereas three studies utilised home visits as one of their strategies to deliver the intervention [36, 38, 39]. Home visits included conducting a monthly comprehensive assessment of known risk factors for frailty at participants' own homes in the community. A comprehensive in-home assessment of known risk factors for frailty was completed during every monthly home visit. During the home visit, referrals to health and social services were also made. Each of the multi-component nurse-led interventions was completed at least once a month with a home visit [38]. Study participants’ homes were visited by a nurse from a community-nursing agency and a nurse conducted an initial and ongoing health assessment. Nurses discussed risk factors for functional decline and provide health education regarding healthy lifestyles during the home visit [39]. Study participants were consulted at a community consultation office by a nurse. A community health nurse performed a comprehensive assessment of the health and well-being, offered tailored advice, and referred to other health professionals as needed during the home visits [36].

### Component of nurse-led intervention

#### Physical exercise

Physical exercise was employed as one of the components of the intervention in three studies. The exercise and physical activities were tailored to the individuals' level of health, preference, and needs [37]. Older people were engaged in upper and lower limb resistance exercises followed by a range of motion exercise as a cool-down. The exercise was performed in groups of eight to ten older people and the Rate of Perceived Exertion (RPE) was measured [37]. An intervention consisting of two 40-min sessions once a week for 12 weeks was employed. A 40-min group exercise session was administered to approximately 10 to 15 participants. To ensure the intervention sessions were engaging and fun, the intervention consisted of stretching, resistance exercises with elastic TheraBands, and aerobic movements on rhythmic music selected by the participants [1]. In another study, physical exercise was used as an individualised intervention for 90 min, twice per week, for 12 weeks, followed by 12 weeks of home exercises. The study participants performed the exercises in groups of eight to ten [40]. They were encouraged to continue daily individualised exercise at their home. In addition to the intervention being provided at the senior centre, participants were also encouraged to perform upper body exercise at their home [37]. Educational leaflets were distributed for study participants who missed the exercise class to guide them when they exercised in their own home [1]. In addition to the group based exercise, participants were encouraged to continue daily individualised exercise assignments at home [40].

#### Nutrition

Nutritional education was employed as an intervention in three studies [1, 37, 40]. One of the three studies [40], employed a person-centered nutritional intervention consisting of nutrition education and counseling tailored to individuals' health status, chronic conditions, diet, household structure, and living environment. Study participants were encouraged to set their own weekly plan to improve their diet [37]. In another study, nutritional health education was provided once per month. Nutrition education or cooking classes focused on selected healthy foods and convenient recipes [1]. Study participants were also provided a commercial formula, iron and folate, vitamin B6, vitamin B12, Vitamin D, and calcium supplement taken daily for 24 weeks [40]. The nutritional intervention was successful in reversing frailty in community-dwelling older people [40]. The mean frailty.

score showed a reduction from 2.1 (0.78) at baseline to 1.5 (1.06) at the 12 months, 95% CI (− 0.92 to − 0.34) [40]. Moreover, compared to the control group, the intervention group who received nutrition (2.98, 95% CI 1.10, 8.07), cognition (2.89, 95% CI 1.07, 7.82), and physical interventions (4.05, 95% CI 1.50, 10.8) showed a statistically significant difference in frailty reduction [40]. However, one study designed for a 12-week multicomponent intervention reported a non-significant frailty reduction over time. The study found that the pre-test, post-test, and the 12-week post-test frailty mean scores were 10.48 (0.47), 8.53 (0.50), and 8.40 (0.52) respectively (*p* <  = 0.46) [1].

#### Cognitive education and training

Interventions focusing on improving cognition was provided by two studies [1, 40]. A cognitive training session for additional 40 min was provided after an exercise session was completed. The session included either calendar making or Cup Nanta alternatively for every other week [1]. Cup Nata was used as a performances involving tapping cups on a desk in harmony designed to strengthen fingers and improve sociality and emotional bonds among participants [1]. In addition, one study included psychosocial intervention through group-based education and counselling on methods to protect and manage mental health including depression and stress. A group-based psychosocial intervention was conducted once per week for about 30 min per session [37]. Interventions focused on cognition was successful in frailty reduction. For the intervention group, the mean frailty score showed a significant reduction over 12 weeks from 1.45 (0.51) at baseline to 0.70 at the follow-up (*P* < 0.01). The components of the interventions, mode of delivery, settings and the rigor of the studies summarised (Table 5).

**Table 5:** Summary of the type of intervention, mode, setting, and rigor of the studies, 2022

Activity	Studies					
	Study 1: (Ha and Park 2020 [37])	Study 2: (Markle-Reid, Browne, and Gafni 2011 [38])	Study 3: (Markle-Reid et al. 2006 [39])	Study 4: (Marcus-Varwijk et al. 2020 [36])	Study 5: (Song and Boo 2022 [1])	Study 6: (Ng et al. 2015 [40])
*Intervention*						
Physical activity	☑	x	x	x	☑	☑
Nutrition education	☑	x	x	x	☑	☑
Counselling and education	☑	¥	✓	☑	x	x
Cognitive training	x	x	x	x	☑	☑
Combination intervention	☑	☑	☑	☑	☑	☑*
*Mode*						
Face-to-face	☑	☑	☑	☑	☑	☑
Telephone support	☑	x	☑	x	x	x
*Setting*						
In-home	x	☑	☑	☑	x	x
Community centre	☑	x	x	x	☑	☑
*Rigor*						
Training provided	x	x	☑	☑	x	x
Follow-up of ≤ 6 months	☑	x	☑	x	x	x
Follow-up of ≤ 12 months	x	☑	x	☑	☑	☑

Activity types specified

^*^Combination of physical exercise, cognitive training and nutrition intervention

^¥^Health plan and goal setting, education about management of illness, and use of empowerment strategies to enhance independence

✓Education regarding healthy lifestyles, and the management of chronic illnesses using a participatory approach

^x^Not mentioned

### Evidence of effectiveness

In the studies, nurse-led interventions were not consistent, but were likely to reverse frailty progression, improve physical functioning, nutritional status, and quality of life, enhance perceptions of social support, improve mental health, and reduce depression.

Reduced frailty, improvements in nutritional status, improved physical performance, and reduction in depression have significant clinical implications for older persons living in the community. These may contribute to maintaining or regaining independence in frail older persons, assist them with activities of daily living, reducing the risk of falls, improved psychological functioning, and improving their overall well-being [45, 46]. Identifying frailty in community-dwelling older people can also have clinical implications to healthcare providers in targeting interventions to improve health-related outcomes including quality of life of community-dwelling frail older people [47].

## Discussion

Frailty is a very important clinical condition, which deserves the attention of healthcare professionals [48]. The current systematic review gathered evidence on the effectiveness of nurse-led interventions in reducing frailty in community-dwelling older people.

### Nurse-led intervention settings

As the population of older people is growing, preventive actions targeting the health of community-dwelling frail older people is becoming increasingly important [49]. Public health nurses have utilised preventive programs to increase the independence of community-dwelling older people through home visits [50]. Preventive home visits appear to help slow down the decline in health-related quality of life (HRQoL) among older people [51]. Frail older people experience health-related problems in multiple domains, including physical, psychological, and social domains, which in-turn decreases their capacity to overcome stress stimuli from acute and chronic illnesses [52]. Older people preferred home visits to focus on their social background and well-being [53]. In the current systematic review, three of the six included studies [36, 38, 39] used home visits by nurses as a strategy to provide the intervention. However, the frequency of home visit was not consistent across the studies. Though the frequency of home visits varies between these studies, those older people who received a regular monthly home visits by nurses showed a reduction in frailty and improvements in quality of life. One study also showed that frail older people who received home visiting showed improvements in psychosocial functioning [54]. Similarly, a systematic review of home visiting interventions has found that community-dwelling older persons with existing disabilities strongly benefit from nurse home visits [55]. A health visit program performed by nurses is feasible and brings a positive outcome for frail older people [21]. A systematic review and meta-analysis also revealed that a well-planned home visit can reduce mortality and admission to institutional care [56].

Studies conducted in community senior centres using a multi-component intervention were effective in reversing frailty among community-living older people [1, 37, 40]. A multi-component intervention approach was effective to manage frailty at the community level [57]. A study also showed that an intervention for the older people in senior centers can help to build resilience and combat frailty in the rapidly ageing society [58].

### Physical exercise

In recent years, regular physical exercise proposed as preventive strategies for frailty and its adverse outcomes [59]. Physical exercise among older people in community-based setting is one of the most effective interventions with systemic effect for improving physical and psychological impairments related to frailty [60]. In the current review, three studies included physical exercise as components of the intervention in their studies. The intervention group received the physical exercise in a variety of techniques across the studies. Studies included in this systematic review showed an improvement in the physical health of the older people [37] and a decrease in frailty level [40]. This finding is supported with studies that stated physical exercise was found beneficial for frailty [61] and can potentially prevent or reverse frailty status of older people [62]. One systematic review also suggested that frail older people appeared to benefit from exercise interventions [63]. However, a study conducted in Korea [1] did not state the effect of physical exercise on the level of frailty among older people.

### Nutrition

Studies found that nutritional status was closely associated with the degree of frailty [64] and malnutrition increases morbidity and mortality of frail older people [65]. As nutrition is a modifiable risk factor for frailty, strategies to prevent and treat frailty should consider nutritional interventions [66]. Nutritional education and protein-energy supplementation showed a reduction in frailty status among older people [67]. In the current review, three studies noted that nutritional interventions either in the form of education, training [1, 37], or nutrition supplementation [40] demonstrated a reduction in frailty among older people living in the community. This finding is in line with a study reported initiatives like nutritional educations as a means of improving the frailty status of community-dwelling older people [68]. However, another study found that nutritional supplements or nutritional education delivered in alone may not be effective for the management of frailty in older people [69]. Studies underlined the need of continued efforts to find effective interventions for community-dwelling frail older people [48].

### Strengths and limitations

The review summarised evidence from nurse-led interventional studies using both randomized and non-randomised trials focussing on community-dwelling older people. Home-based programs are more accessible, feasible and would eliminate the barrier of transportation for many frail older people living in community settings. A number of studies did not provide sufficient detail on their method of assessing frailty and improving measures, which may limit the validity of our findings. Further studies incorporated cost- analysis would be beneficial to conclude for the overall effectiveness of multi-component nurse-led frailty interventions. Moreover, research articles published in languages other than English were not considered. Consequently, a narrower range of perspectives may be revealed, which can potentially limit the research's conclusions.

## Conclusion

Data were obtained from methodologically appraised studies. Two of the quasi-experimental and one of the RCT studies showed a moderate methodological quality. Given the non-randomized study designs and moderate methodological quality of certain included studies, it is imperative to recognise the significance of designing a multifaceted intervention that addresses the multiple underlying factors associated with frailty among community-dwelling older individuals. Evaluating physical functioning, nutritional status, mental health, social support, and quality of life in community-dwelling frail older people can contribute to develop appropriate interventions to promote healthy ageing, independence, overall well-being, and improve quality of life. Future research needs to focus on developing clear and consistent definitions and measures of frailty, as well as exploring the long-term effect of interventions on frailty outcomes.

### Implication to practice

Evidence-based nurse-led intervention models promote the health of frail older people and reduce its unwanted long-term consequences. When assessing frail older people in the community, it is imperative to assess depressive symptoms, HRQOL, and related physical and psychosocial health outcomes.

### Implication to research

To evaluate the effectiveness of nurse-led interventions, further studies are needed that consider the use of standardised screening tools, the settings and designs, and the cost-effectiveness of the intervention.

### Ethics

As this project is a systematic review, ethical approval is not required. Patients or the public were not involved in the development of this review.

### Supplementary Information

**Additional file 1.** PRISMA 2020 checklist.

**Additional file 2.** Full search strategies for the databases utilised, 2022.

**Additional file 3.** List of excluded studies and reasons for exclusion (*n*=12).

## Data Availability

All data generated or analysed during this study are included in this manuscript.
