# Supplementary material for: The effectiveness of nurse-led interventions to manage frailty in community-dwelling older people: a systematic review
Source: Syst Rev. 2023 Sep 30;12:182. doi: 10.1186/s13643-023-02335-w (PMC10543273; doi:10.1186/s13643-023-02335-w)
Supplement: Supplementary file 2 — Additional file 2. Full search strategies for the databases utilised, 2022. [file 13643_2023_2335_MOESM2_ESM.docx]

**Appendix 2: Full search strategies for the databases utilised, 2022**

| **Database** | **Search strings** | **Number of articles** |
| --- | --- | --- |
| PubMed | ((((nurse-led OR "nurse led") AND (education OR training OR intervention OR program OR teaching)) AND (frail OR fragile OR "frailty syndrome" OR debility OR infirmity)) AND (elder OR aged OR old OR geriatric)) AND (("community based settings" OR "community-based" OR "community setting" OR community) | 29 |
| Medline | nurse-led OR "nurse led" ) AND ( education OR training OR intervention OR program OR teaching ) AND ( frail* OR fragile* OR "frailty syndrome" OR debility OR infirmity ) AND ( elder* OR aged* OR old* OR geriatric ) AND ( ("community based settings" OR "community-based" OR "community setting" OR community ) | 26 |
| CINAHL | ( nurse-led OR "nurse led" ) AND ( education OR training OR intervention OR program OR teaching ) AND ( frail* OR fragile* OR "frailty syndrome" OR debility OR infirmity ) AND ( elder* OR aged* OR old* OR geriatric ) AND ( ("community based settings" OR "community-based" OR "community setting" OR community ) | 23 |
| Web of Science | [**((((ALL=(nurse-led OR "nurse led")) AND ALL=(education OR training OR intervention OR program OR teaching)) AND ALL=(frail* OR fragile* OR "frailty syndrome" OR debility OR informity)) AND ALL=(elder* OR aged* OR old* OR geriatric )) AND ALL=("community based settings" OR "community-based" OR "community setting" OR community)** \|](https://www-webofscience-com.ezproxy.uow.edu.au/wos/woscc/summary/587ef5fb-7bec-4939-805a-4ef629a36ac1-41a72063/relevance/1) | 36 |
| Scopes | ( TITLE-ABS-KEY ( nurse-led OR {nurse led} ) AND TITLE-ABS-KEY ( education OR training OR intervention OR program OR teaching ) AND TITLE-ABS-KEY ( frail* OR fragile* OR {frailty syndrome} OR debility OR infirmity ) AND TITLE-ABS-KEY ( elder* OR aged* OR old* OR geriatric ) AND TITLE-ABS-KEY ( {community based settings} OR {community-based} OR {community setting} OR community ) ) | 28 |
| PsychInfo | ( nurse-led OR "nurse led" ) AND ( education OR training OR intervention OR program OR teaching ) AND ( frail* OR fragile* OR "frailty syndrome" OR debility OR infirmity ) AND ( elder* OR aged* OR old* OR geriatric ) AND ( "community based settings" OR "community-based" OR "community setting" OR community ) | 9 |
| WHO Global Index Medicus | (tw:(nurse-led OR "nurse led")) AND (tw:(education OR training OR intervention OR program OR teaching)) AND (tw:(frail OR fragile OR "frailty syndrome" OR debility OR infirmity)) AND (tw:(elder OR aged OR old OR geriatric)) AND (tw:(community based settings" OR "community-based" OR "community setting" OR community)) | No document |
|  | Other sources | 5 |
| Total |  | 156 |
