# Supplementary material for: The effectiveness of nurse-led interventions to manage frailty in community-dwelling older people: a systematic review
Source: Syst Rev. 2023 Sep 30;12:182. doi: 10.1186/s13643-023-02335-w (PMC10543273; doi:10.1186/s13643-023-02335-w)
Supplement: Supplementary file 3 — Additional file 3. List of excluded studies and reasons for exclusion (n=12). [file 13643_2023_2335_MOESM3_ESM.docx]

Appendix 3: List of excluded studies and reasons for exclusion (n=12).

| **Reference** | **Reason for exclusion** |
| --- | --- |
| Salem BE., et al. Constructing and identifying predictors of frailty among homeless adults—A latent variable structural equations model approach. Arch Gerontol Geriatr. 2014 Mar-Apr;58(2):248-56. doi: 10.1016/j.archger.2013.09.005. PMID: 24505611; PMCID: PMC4005873. | Not an interventional study. |
| Marsden E., et al. A structure, process and outcome evaluation of the Geriatric Emergency Department Intervention model of care: a study protocol. BMC Geriatr. 2017 Mar 23;17(1):76. doi: 10.1186/s12877-017-0462-z. PMID: 28330452; PMCID: PMC5363028. | Not an interventional study. |
| Grouw YL, Bannink d., and Van Hout H. Care Professionals Manage the Future, Frail Older Persons the Past. Explaining Why Frailty Management in Primary Care Doesn't Always Work. Geriatric Medicine  Volume 7 - 2020 \| <https://doi.org/10.3389/fmed.2020.00489> | Not an interventional study. |
| Allen KR., The after-discharge care management of low-income frail elderly (AD-LIFE) randomized trial: theoretical framework and study design. Popul Health Manag. 2011 Jun;14(3):137-42. doi: 10.1089/pop.2010.0016. Epub 2011 Feb 15. PMID: 21323461; PMCID: PMC4971414. | Not community-based: A theoretical framework to assess after-discharge care management. |
| Hullick C, Conway J, Higgins I, Hewitt J, Dilworth S, Holliday E, Attia J. Emergency department transfers and hospital admissions from residential aged care facilities: a controlled pre-post design study. BMC Geriatr. 2016 May 12;16:102. doi: 10.1186/s12877-016-0279-1. PMID: 27175921; PMCID: PMC4866019. | Not community-based |
| Stijnen MM, Jansen MW, Duimel-Peeters IG, Vrijhoef HJ. Nurse-led home visitation programme to improve health-related quality of life and reduce disability among potentially frail community-dwelling older people in general practice: a theory-based process evaluation. BMC Fam Pract. 2014 Oct 25;15:173. doi: 10.1186/s12875-014-0173-x. PMID: | A theory-based process evaluation did not deliver an intervention. |
| Bleijenberg N, Drubbel I, Neslo RE, Schuurmans MJ, Ten Dam VH, Numans ME, de Wit GA, de Wit NJ. Cost-Effectiveness of a Proactive Primary Care Program for Frail Older People: A Cluster-Randomized Controlled Trial. J Am Med Dir Assoc. 2017 Dec 1;18(12):1029-1036.e3. doi: 10.1016/j.jamda.2017.06.023. Epub 2017 Aug 8. PMID: 28801235. | Not focused on frailty improvement: A cost-effectiveness analysis: |
| Craig L. The role of the registered nurse in supporting frailty in care homes. Br J Nurs. 2019 Jul 11;28(13):833-837. doi: 10.12968/bjon.2019.28.13.833. PMID: 31303040. | Not community-based and doesn’t report the intended outcome. |
| Zhao M, Yang Z, Wang Y, Li M, Wang K. Resident- and Institutional-Level Factors, Frailty, and Nursing Homes Residents. Nurs Res. 2022 Jan-Feb 01;71(1):E1-E9. doi: 10.1097/NNR.0000000000000556. PMID: 34620773. | Not community-based |
| Lyndon H, Latour JM, Marsden J, Kent B. Designing a nurse-led assessment and care planning intervention to support frail older people in primary care: An e-Delphi study. J Adv Nurs. 2022 Apr;78(4):1031-1043. doi: 10.1111/jan.15066. Epub 2021 Oct 8. PMID: 34626001; PMCID: PMC9291776. | Not community-based |
| Garcia-Canton C, Rodenas A, Lopez-Aperador C, Rivero Y, Anton G, Monzon T, Diaz N, Vega N, Loro JF, Santana A, Esparza N. Frailty in hemodialysis and prediction of poor short-term outcome: mortality, hospitalization and visits to hospital emergency services. Ren Fail. 2019 Nov;41(1):567-575. doi: 10.1080/0886022X.2019.1628061. PMID: 31234684; PMCID: PMC6598473. | Not community-based |
| Smoliner C, Norman K, Scheufele R, Hartig W, Pirlich M, Lochs H. Effects of food fortification on nutritional and functional status in frail elderly nursing home residents at risk of malnutrition. Nutrition. 2008 Nov-Dec;24(11-12):1139-44. doi: 10.1016/j.nut.2008.06.024. Epub 2008 Sep 11. PMID: 18789649. | Not community-based |
